# Enhanced Transcriptional Activation in Developing Mouse Photoreceptors

**DOI:** 10.1167/iovs.66.1.54

**Published:** 2025-01-24

**Authors:** Brendon M. Patierno, Mark M. Emerson

**Affiliations:** 1 Biology and Biochemistry PhD Programs, Graduate Center, City University of New York, New York, New York, United States; 2 Department of Biology, The City College of New York, City University of New York, New York, New York, United States

**Keywords:** *cis*-regulatory elements, photoreceptors, gain of function, loss of function, in vivo

## Abstract

**Purpose:**

Retinal development in the mouse continues past birth and provides a widely used model system in which photoreceptor formation can be observed and manipulated. This experimental paradigm provides opportunities for both gain-of-function and loss-of-function studies, which can be accomplished through in vivo or ex vivo plasmid delivery and electroporation. However, the *cis*-regulatory elements used to implement this approach have not been fully evaluated or optimized for the unique transcriptional environment of photoreceptors.

**Methods:**

Here we investigate whether the use of a photoreceptor *cis*-regulatory element from the *Crx* gene in combination with broadly active promoter elements can increase the targeting of developing photoreceptors in the mouse. This study characterizes the in vivo activity of this element for the first time, as well as explores its use as a tool for gain-of-function and loss-of-function experiments.

**Results:**

We report that a *cis*-regulatory element from the *Crx* gene, in combination with broadly active promoter elements, increases the targeting of developing rod photoreceptors in the mouse. Additionally, the same element can be used to target developing cones at embryonic time points by ex vivo electroporation. Utility of this combined element includes greater reporter expression, as well as enhanced overexpression and loss-of-function phenotypes in photoreceptors.

**Conclusions:**

This study highlights the importance of identifying and testing relevant *cis*-regulatory elements when planning cell subtype-specific experiments. The use of specific hybrid elements will provide a more efficacious gene delivery system to study mammalian photoreceptor formation, which will benefit research with potential therapeutic relevance for blinding diseases.

The vertebrate retina is a complex tissue that contains many cell types that must function together in precise organization. During development, these cell types differentiate after formation from retinal progenitor cell (RPC) division and orient within the retina appropriately. Differentially open chromatin regions of genomic DNA and cell type–specific transcription factor expression within these cells define which gene regulatory networks are active in postmitotic cells as opposed to RPCs, and differences exist even within cell subtypes. Thus, knowledge of cell type–specific transcription factors and their corresponding *cis*-regulatory elements (CREs) is critical to studying the development of the retina.

The developing retina is highly amenable to experimentation that implements gain-of-function or loss-of-function approaches through introduction of exogenous vectors, such as in plasmid electroporation. However, these approaches rely on sequences in the introduced vectors to promote transcription within very different cell types that can also be in varied stages of differentiation. It is therefore important to understand which cell types are efficiently targeted by the transcription activating sequences present in the introduced vectors.

Several commonly used vectors for ubiquitous overexpression include previously identified transcriptional elements such as CAG, EF1A, and CMV that were defined in nonretinal tissues. Differences in the promoter activity of these elements in retina cells were first observed in rat tissue with the observation that CAG was the most active element in photoreceptors.^1^ Subsequent studies in the developing mouse retina thus have most often used CAG as a ubiquitous promoter and qualitatively observed CAG::GFP to have similar activity in vivo to what was seen in the rat retina.^2^ However, upon examination of postnatal retina experiments that use the CAG element, we observed that CAG-driven reporter activity in newly differentiating photoreceptors is often sparse.^3^ Previous research has suggested the plasticity of photoreceptor precursor cells, which can be driven toward rod, S cone, or M cone fates based on manipulations to *Nrl* and *Thrb* at this developmental stage.^4^ Therefore, it is crucial to determine whether CAG is the most appropriate plasmid element to drive transcription in these developing cells, which are often the target of photoreceptor fate change manipulations. Indeed, the effectiveness of CAG to drive transcription in developing photoreceptors has not been systematically tested, and CAG-driven reporter plasmids often appear to underrepresent photoreceptors when co-electroporated with reporters driven by photoreceptor active elements.^3^ We have observed sparser activity of CAG in developing rod photoreceptors compared to other cell types but highlight this phenomenon for the first time here using a photoreceptor-active CRE located approximately 1500 base pairs downstream of the *Crx* gene.

The *Crx* gene is expressed during both cone and rod genesis, and its expression in mouse and chick retina tissue has been characterized as highly expressed in postmitotic photoreceptor cells.^5^^–^^8^ *Crx* expression is, however, also seen to a lesser degree in bipolar cells,^8^ underscoring the importance of identifying the CREs that regulate this gene in different cell types. The specific CRE further characterized in this study, CrxE1, was first identified and predicted to be photoreceptor specific based on a high number of NRL, NR2E3, and CRX binding sites and through its ability to drive a reporter in developing mouse rods.^9^ This element was further characterized in both chick and mouse retinal tissue, and while still mostly active in photoreceptors, it was also observed in a population of bipolar cells by ex vivo assay.^3^ However, the activity of this element has not yet been characterized in vivo in mouse tissue. Taking this information together, CrxE1 may be one of the earliest CREs active in newborn photoreceptor cells and requires further characterization.

Although changes in photoreceptor cell proportions and opsin expression phenotypes have been observed through various gain-of-function and loss-of-function experiments, it has not been explored whether these approaches could be optimized by the use of alternative or additional enhancer elements. The observed phenotype generated by using plasmid-based overexpression of *Onecut1* (OC1) to induce cone photoreceptor gene expression programs in postnatal mice is of particular interest.^10^ Similarly, the knockdown of *Nrl* at p0 using CRISPR/Cas9-based vectors has been shown to drive the induction of the shortwave-sensitive opsin protein associated with blue light transducing cones.^11^ While the genes responsible have been the subject of thorough characterization, there is little known in the field regarding how photoreceptor-specific CREs could be used to enhance the degree to which these phenotypes are observed. Such knowledge could be critical for the generation of cone cells in other potentially clinically relevant models, such as human retinal organoids or induced pluripotent stem cells. Therapeutic strategies that rely on transforming retinal cells before transplantation would benefit from an increase in the number of cells that are efficiently transformed.

This study characterizes CrxE1 activity in the mouse postnatal retina in vivo for the first time. Additionally, this study explores the method of combining cell type–specific enhancers with broadly active promoters in plasmid delivery systems, including using this method to target more developing cones embryonically by ex vivo electroporation. The observation that the CrxE1 element is active in cells where CAG is not yet active or is weakly active led to the hypothesis that there is potential to increase the number of cells that can be targeted by ubiquitous promoter-based systems to include more developing photoreceptors. Currently, these experiments are frequently performed using plasmids that contain only broadly active promoters such as CAG or EF1A. The results of this study demonstrate that the inclusion of the CrxE1 element with these more broadly active elements represents a powerful tool to target developing photoreceptors in the mouse retina.

## Methods

### Animals

All procedures involving animals were approved and conducted in accordance with the City College of New York Institutional Animal Care and Use Committee–approved protocols and the ARVO Statement for the Use of Animals in Ophthalmic and Vision Research. CD-1 mice were obtained from Charles River (Kingston, NY, USA).

### Molecular Biology

CrxE1::GFP^3^ (referred to as CrxEnh1 in Emerson et al.^10^), which was derived from the sequence identified by Hsiau et al.^9^; CAG::TdTomato^12^; CAG::mCherry^13^; CAG::OC1^10^; and empty vectors, including Stagia^14^ and modified px458,^15^ have been previously described. The CrxE1/CAG::GFP element was made using cloning strategies described here. The CrxE1/CAG::OC1 element was made using PCR with the high-fidelity Herculase II Fusion DNA Polymerase (cat. 600675; Agilent [Santa Clara, CA, USA]) to add “Sal1” sites to the 261-bp enhancer sequence and then using said sites to ligate the fragment into CAG::OC1. A version of px458 that contains the EF1A promoter,^16^ including variants with nuclear-localized versions of GFP or mCherry, was a gift from Joseph Brzezinski (plasmids 159654, 159655; Addgene [Watertown, MA, USA]). The GFP version of the plasmid was modified to include CrxE1 immediately upstream of the EF1A sequence using Sal1 sites. To maintain the unique Bbs1 sites in the px458 vector for insertion of guide sequences, a Bbs1 restriction site in the CrxE1 sequence was mutated by one base pair (GTCTTC to GACTTC). The previously reported “*Nrl* guide 2”^11^ was then cloned into the base (EF1A) px458 vector as well as the modified CrxE1/px458 vector using Bbs1. All constructs generated through PCR were sequence verified.

### Electroporation

Ex vivo electroporation experiments were performed as previously reported^17^ with the following modifications. The electroporation chamber was rinsed with 70 µL 1× PBS for a minimum of eight times between groups. Electroporation mixes were made using 50 µL total volume in a 1× PBS final concentration, with plasmid DNA for each construct added to 10 µg total (0.2 µg/µL). For ex vivo electroporation, the electroporator parameters were 25-volt pulses, a pulse length of 50 ms, pulse intervals of 950 ms, and five pulses total.

In vivo electroporations were performed as previously reported^1^ with the following modifications. Pulled glass needles (1B100F-4; World Precision Instrument [Sarasota, FL, USA]) were backfilled with each mix before electroporation. In vivo electroporation mixes were made using a total of 10 µL volume in Tris-EDTA (TE), with each plasmid DNA added to 10 µg total (1 µg/µL). For electroporation mixes using two plasmids, 10 µg of each were used. A Femtojet (Eppendorf [Hamburg, Germany]) was used to inject DNA into the subretinal space. The volume injected was qualitatively measured by the appearance of the FastGreen dye filling the subretinal space and was approximately 0.1 to 0.2 µL per retina. Tweezer-type electrodes (CUY650P-10; Bulldog Bio [Portsmouth, NH, USA]) were used to apply electrical pulses. A Nepagene NEPA21 Type II Super Electroporator (NEPA21; Bulldog Bio) was used for both ex vivo and in vivo electroporations. For in vivo electroporation, the electroporator parameters were 80-volt pulses, a pulse length of 50 ms, interpulse intervals of 950 ms, and five pulses total. Control and experimental mice from a litter were tattooed at the time of electroporation so as to identify them at the time of retinal harvest.^18^

### Ex Vivo Explant Culture and Fixation

Retinae were cultured on 13-mm/0.2-micron floating filters (10417001; Cytiva [Marlborough, MA, USA]) on Dulbecco's modified Eagle's medium/F-12 media (11320082; Gibco [Waltham, MA, USA]) with 10% fetal bovine serum (Thermo Fisher Scientific [Waltham, MA, USA], A3160602) and 1× L-glutamine, penicillin, and streptomycin (10378016; Gibco). Retinae were fixed for 30 minutes at room temperature in 4% paraformaldehyde in 1× PBS.

### Immunofluorescence

Primary antibodies were diluted in a 1× PBS with 0.3% Triton X-100 solution. Primary antibodies used were rabbit anti-GFP 1:500 (A-6455; Thermo Fisher Scientific), mouse anti-RXRG 1:30 (SC-365252; Santa Cruz Biotechnology, Dallas, TX, USA), mouse anti–RHODOPSIN 1:500 (SC-57432; Santa Cruz Biotechnology), goat anti-Opn1SW 1:1000 (SC-14363; Santa Cruz Biotechnology), rabbit anti-RFP 1:250 (600-401-379; Rockland Immunochemicals [Philadelphia, PA, USA]), and rabbit anti-ONECUT1 1:300 (25137-I-AP; Proteintech [Rosemont, IL, USA]). Rabbit anti-GFP was strictly used to enhance GFP signal from px458-based vectors and was not used in any other conditions. All secondary antibodies were obtained from Jackson Immunoresearch (West Grove, PA, USA) and were designated as appropriate for multiple labeling. Retinae were processed for staining as previously described.^17^

### Microscopy

All confocal images were obtained using a Zeiss 800 confocal microscope (Oberkochen, Germany). Images were analyzed using ImageJ (National Institutes of Health, Bethesda, MD, USA) and displayed using Affinity Designer software (Nottingham, UK). All quantified images were analyzed blinded to the experimental condition. Files were duplicated and saved without identifiers, selected randomly, quantified, and then matched to their original file name.

### Statistical Analysis

When testing for statistically significant differences between groups, nested *t*-tests were performed using GraphPad (GraphPad Software, La Jolla, CA, USA). While the three measurements within a subcolumn of our data are from different sections cut from the same retina, each set of three measurements within a group is from different retinae. Considering this third variable is present, we chose to perform a nested *t*-test as recommended by the “Analysis Checklist” provided by GraphPad. This test performs a standard unpaired *t*-test between groups while also reporting variance data within groups.

## Results

### CrxE1 Activity in Postnatal Mice Is Photoreceptor Specific

**Figure 1.**
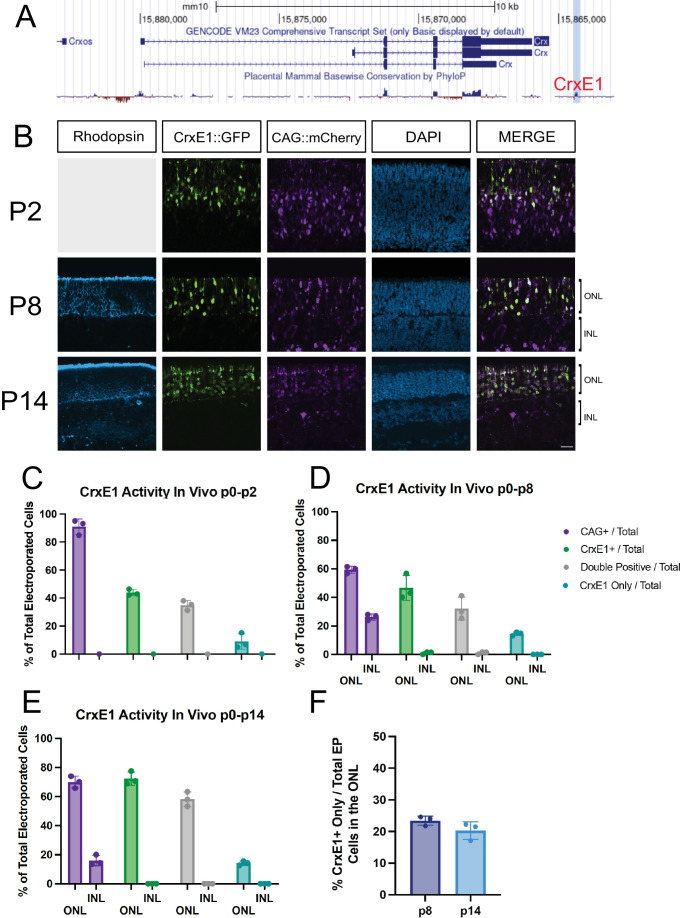
Characterization of CrxE1::GFP activity compared to CAG::mCherry activity across developmental time points. (**A**) Location of the 243-bp CrxE1 sequence in relation to the *Crx* gene. Snapshot is taken from the UCSC genome browser with placental mammalian conservation tracks shown below. The *cis*-regulatory element is identified by *red font* and the location highlighted with *light blue shading*. (**B**) Confocal microscopy of retinal sections electroporated in vivo at p0 and processed at p2, p8, or p14. Representative z-projections are shown with the developing ONL at the top of the section. Columns from *left* to *right* are RHODOPSIN immunostaining in *cyan*, CrxE1::GFP epifluorescence, CAG::mCherry epifluorescence, 4′,6-diamidino-2-phenylindole staining in *blue*, and a merge of the CrxE1 and CAG reporter channels (**C–E**). Quantification of reporter activity across three biological samples using total electroporated cells as the denominator and categorized for ONL or INL for panels **C** and **D** when the INL is apparent (**F**). Quantification of CrxE1^+^-only cells out of electroporated cells specifically in the ONL as defined by RHODOPSIN immunostaining. *Scale bar*: is 20 µm in panel **A** and applies to all panels. EP, electroporated; GCL, ganglion cell layer.

To determine the activity of the CrxE1 element in the mouse retina in vivo, retinal electroporation was performed at p0 with a CrxE1::GFP reporter plasmid as well as a CAG::mCherry reporter plasmid (Fig. 1). A minimum of three retinae, each from separate individuals, were dissected and fixed at p2, p8, and p14. All retinae were stained with 4′,6-diamidino-2-phenylindole, and for retinae harvested at p8 and p14, a RHODOPSIN antibody was used to visualize rod photoreceptor cells in the outer nuclear layer (ONL). The CrxE1 element was shown to drive activity specifically in the ONL, with only an occasional cell that appeared displaced into the inner nuclear layer (INL). At p8, 97.4% ± 2.2% of GFP^+^ cells were in the ONL, and at p14, 99.6% ± 0.6% of GFP^+^ cells were in the ONL (Supplementary Table S1). Analysis of sections by cell counting quantified an average of 9.02% CrxE1^+^-only cells out of total electroporated cells at p2, 14.46% at p8, and 14.12% at p14 (Fig. 1). Virtually every CrxE1^+^-only cell was positioned in the ONL and costained with RHODOPSIN. Conversely, as the division between the INL and ONL becomes more apparent at p8 and p14, a significant portion of the CAG^+^-only cells were clearly located in the INL. Thus, targeting the CrxE1^+^ population in addition to the CAG^+^ one might lead to increased reporting of photoreceptor cells.

### Addition of the CrxE1 to a Broadly Active Promoter Increases Reporter Activity

**Figure 2.**
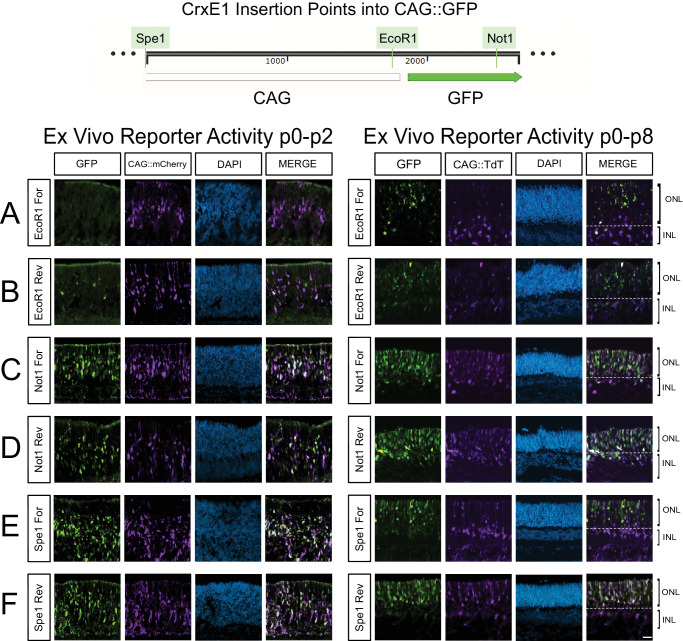
Differential CrxE1/CAG::GFP activity based on insertion point of CrxE1 into the CAG::GFP vector. Retinae were dissected and electroporated ex vivo and cultured for either 2 days or 8 days. Representative z-projections are shown with the developing ONL at the top of the section. (**A**, **B**) Insertion of the CrxE1 element into the EcoR1 site of CAG::GFP in the forward or reverse orientation, respectively. (**C**, **D**) Insertion of the CrxE1 element into the Not1 site of CAG::GFP in the forward or reverse orientation, respectively. (**E**, **F**) Insertion of the CrxE1 element into the Spe1 site of CAG::GFP in the forward or reverse orientation, respectively. Columns from *left* to *right* are GFP epifluorescence driven by various constructs, epifluorescence by either CAG::mCherry at p2 or CAG::TdT at p8, 4′,6-diamidino-2-phenylindole staining in *blue*, and a merge of the GFP and CAG reporter channels. *Scale bar*: 20 µm and applies to all panels.

The observation that as early as p2, the CrxE1 element is active in more precursor or developing photoreceptor cells than CAG (Fig. 1) led to the hypothesis that there is the potential to increase the number of reporter-positive cells within the electroporated population. We constructed a new single plasmid reporter that implemented both the widely used CAG element and the CrxE1 sequence to drive GFP. We tested multiple possible insertion points of CrxE1 into the CAG::GFP vector: one position located upstream of CAG, another between CAG and the GFP coding sequence, and an insertion point in the 3′UTR region of GFP. To screen for reporter activity, we co-electroporated p0 retinae ex vivo with one of the new CrxE1/CAG GFP constructs and a control construct with CAG driving mCherry for p2 time points or TdTomato (TdT) for p8 time points and then cultured the retina for either 2 days or 8 days (Fig. 2; mCherry was chosen for its faster protein maturation time to minimize reporter detectability as a variable at the P2 time point).^19^ It was immediately apparent that insertion of the CrxE1 sequence just 5′ of the GFP element in the plasmid did not increase activity but actually led to the reporting of fewer cells than the original CAG::GFP vector (Figs. 2A, 2B). However, the other insertion points upstream of CAG or downstream of GFP increased the number of GFP^+^ cells proportional to the red fluorescent protein^+^ cells (Figs. 2C–F). There was no consistent observable difference based on the orientation of the sequence in these positions. Insertion of the CrxE1 element just 5′ of the CAG element most consistently increased the number of cells targeted above the CAG-alone condition, and by p8, we observed that GFP^+^-only cells localized in the ONL (Fig. 2E).

### The CrxE1/CAG Element Enhances the Onecut1 Gain-of-Function Phenotype

**Figure 3.**
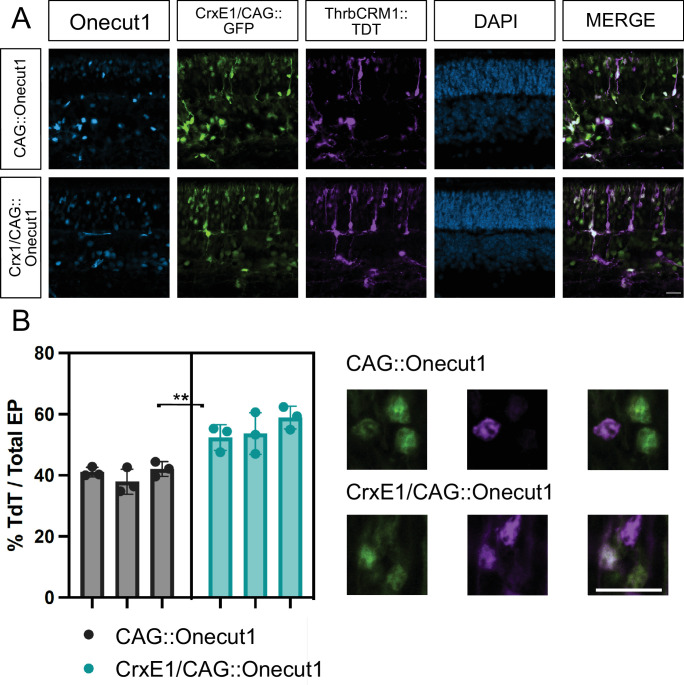
(**A**) Confocal microscopy of retinal sections electroporated in vivo at p0 with CrxE1/CAG::GFP and either CAG::Onecut1 or CrxE1/CAG::Onecut1. Retinae were processed at p14, and sections were stained with a Onecut1 antibody. Representative z-projections are shown with the developing ONL at the top of the section. Columns from *left* to *right* are Onecut1 immunostaining in *cyan*, CrxE1/CAG::GFP epifluorescence, ThrbCRM1::TdT epifluorescence, 4′,6-diamidino-2-phenylindole staining in *blue*, and a merge of the GFP and TdT reporter channels. (**B**) Quantification of ThrbCRM1::TdT reporter and GFP double-positive cells across three biological samples using CrxE1/CAG::GFP-positive cells as the denominator. The statistical method used was the two-tailed nested *t*-test analysis performed within the software program Prism. *P < 0.05, **P < 0.01. *Scale bar*: 20 µm in panel **A** and applies to all panels.

After identification of the CrxE1/CAG::GFP reporter, which qualitatively appeared to increase the amount of photoreceptor cells targeted at p0, we next sought to quantify this increase in plasmid activity. We decided to test whether a similar CrxE1/CAG plasmid vector could be used to drive the expression of a gene of interest more effectively than plasmids using CAG alone. We electroporated p0 mouse retinae in vivo with either a newly constructed CrxE1/CAG::OC1 plasmid or CAG::OC1, as well as our CrxE1/CAG::GFP reporter. Additionally, we included the ThrbCRM1::TdT reporter, which is active in RPCs that generate cones and is a direct target of the OC1 transcription factor. It has already been reported that the ThrbCRM1 element is activated in the postnatal retina in response to *Onecut1* overexpression.^10^ We observed that as early as p14, an average of 40.36% of electroporated cells showed induced ThrbCRM1 activity in the CAG::OC1 group, whereas an average of 55.00% of electroporated cells showed induced ThrbCRM1 activity in the CrxE1/CAG::OC1 group (Fig. 3). We performed a two-tailed nested *t*-test analysis on the data generating this 14.6% ± 2.34% difference between groups and concluded a significant difference with a *P* value of 0.0033. There was no significant difference between technical replicates across samples within a group (Supplementary Table S4).

### CrxE1 Addition Enhances a Cas9 Loss-of-Function Phenotype

**Figure 4.**
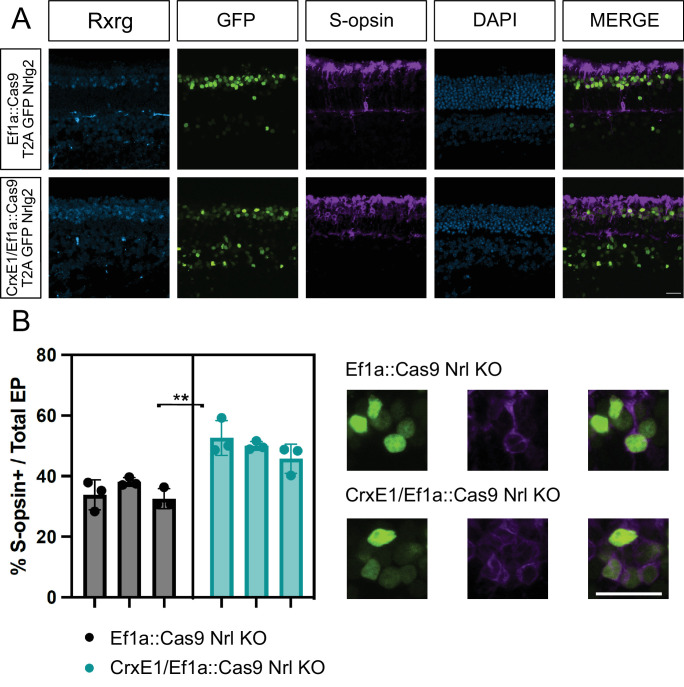
(**A**) Confocal microscopy of retinal sections electroporated in vivo at p0 with either EF1A::Cas9 T2A GFP Nrlg2 or CrxE1/EF1A::Cas9 T2A GFP Nrlg2. Retinae were processed at p21, and sections were stained with an S-opsin antibody. Representative z-projections are shown with the developing ONL at the top of the section. Columns from *left* to *right* are RXRG immunostaining in *cyan*, GFP epifluorescence, S-opsin immunostaining, 4′,6-diamidino-2-phenylindole staining in *blue*, and a merge of the GFP and TdT reporter channels. (**B**) Quantification of S-opsin and GFP double-positive cells across three biological samples using GFP-positive cells as the denominator. The statistical method used was the two-tailed nested *t*-test analysis performed within the software program PRISM. *P < 0.05, **P < 0.01. *Scale bar*: 20 µm in panel **A** and applies to all panels.

Additionally, we wanted to determine whether the CrxE1 sequence could enhance gene editing in the retina. We tested this in the context of the px458 vector system and used a guide that had been validated to work in the mouse retina.^11^ Using a modified px458 vector with the ubiquitous promoter EF1A driving Cas9 and GFP, we inserted the CrxE1 element 5′ of the EF1A promoter to test whether it would increase the efficiency of gene editing in photoreceptors. To assess this, we focused on the *Nrl* transcription factor expressed by rod photoreceptors. When the px458 gene editing plasmid containing a guide RNA targeting *Nrl* was introduced in vivo at p0, rod cells showed an upregulation of S-OPSIN at p21, consistent with the *Nrl* germline knockout phenotype.^11^ We used one of the *Nrl* targeting guide RNAs previously reported to be effective.^11^ In this system, retinae were electroporated with either the EF1A::Cas9 T2A GFP Nrlg2 (px458::Nrlg2) or CrxE1/EF1A::Cas9 T2A GFP Nrlg2 (CrxE1/px458::Nrlg2) plasmids in vivo at p0 and then analyzed at p21. Consistent with the original report using this guide, we observed that at p21, 34.83% of all targeted cells activated S-OPSIN in response to the *Nrl* CRISPR condition in the px458::Nrlg2 group. By comparison, 49.47% of targeted cells activated S-OPSIN in the CrxE1/px458::Nrlg2 group (Fig. 4^5^). We performed a two-tailed nested *t*-test analysis on this 14.6% ± 2.59% difference between groups and identified a significant difference with a *P* value of 0.0049. There was no significant difference between technical replicates across samples within a group (Supplementary Table S5).

**Figure 5.**
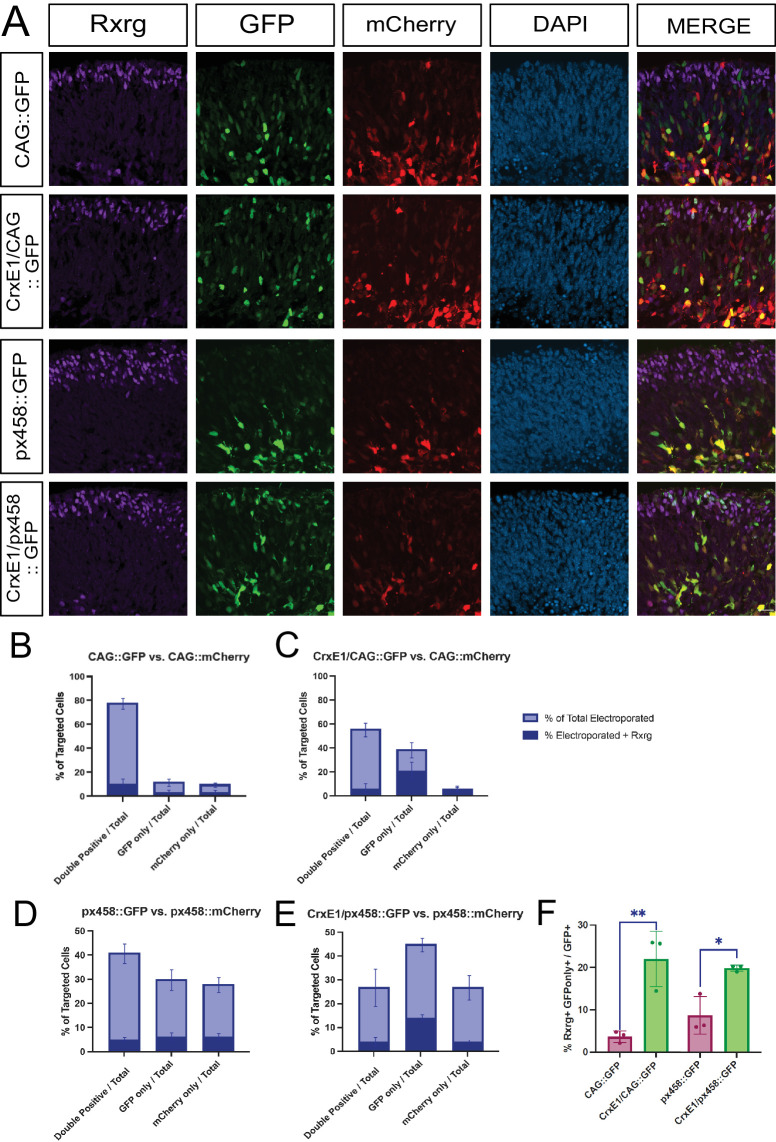
(**A**) Confocal microscopy of retinal sections electroporated ex vivo at E13.5 and cultured for 3 days. Retinas in the first two groups (rows 1 and 2) were electroporated with CAG::mCherry and either CAG::GFP or CrxE1/CAG::GFP. Retinas in the following two groups (rows 3 and 4) were electroporated with EF1A::Cas9 T2A mCherry (px458::mCherry) and either EF1A::Cas9 T2A GFP (px458::GFP) or CrxE1/EF1A::Cas9 T2A GFP (CrxE1/px458::GFP). All retinas were processed after 3 days in culture and stained with RXRG. Columns from *left* to *right* are RXRG immunostaining in *violet*, GFP epifluorescence, mCherry epifluorescence, 4′,6-diamidino-2-phenylindole staining in *blue*, and a merge of the GFP and mCherry reporter channels. (**B–E**) Quantification of the proportion of electroporated cells either GFP^+^, mCherry^+^, or double positive in each group, including the proportion of RXRG^+^ cells within each subset. (**F**) Quantification of RXRG and GFP only out of the total GFP^+^ population. We performed a two-tailed unpaired *t*-test analysis between the relevant experiment groups. *P < 0.05, **P < 0.01. *Scale bar*: 20 µm in panel **A** and applies to all panels.

### CrxE1 Addition Enhances Targeting of Rxrg-Positive Cells at E13.5

Finally, we were interested in testing both the CrxE1/CAG::GFP and the CrxE1/EF1A::Cas9 T2A GFP plasmids for whether they could increase the transcriptional targeting of cone photoreceptors. To interrogate the reporter activity of CrxE1/CAG::GFP in cones, E13.5 mouse retinae were electroporated ex vivo with CAG::mCherry and either CAG::GFP or CrxE1/CAG::GFP. The retinae were then cultured for 3 days and harvested, and slides were stained with RXRG antibody (Fig. 5). RXRG is expressed in cone photoreceptors but also in retinal ganglion cells (RGCs).^20^ As there is a clear spatial separation between these two cell subtypes, cells below the more apical half of the retina were considered RGCs and not considered RXRG positive for the sake of this assay. Colocalization of GFP activity with cells expressing endogenous RXRG were indicative of a cone photoreceptor cell targeted by the reporter plasmid. We observed an increase in the proportion of GFP^+^-only cells out of the total electroporated population from an average of 12.6% to 38.6% when comparing the group with CAG::GFP to the group with CrxE1/CAG:GFP (Supplementary Table S6). The proportion of RXRG and GFP-only double-positive cells out of the total GFP^+^ population increased from an average of 3.66% to 22.99%. Following the same procedure, we compared the reporter activity of CrxE1/EF1A::Cas9 T2A GFP (CrxE1/px458::GFP) to the activity of EF1A::Cas9 T2A GFP (px458::GFP) at E13.5. Mouse retinae were electroporated with a modified version of the px458 vector containing mCherry and either CrxE1/px458::GFP or px458::GFP. We observed an increase in the proportion of GFP^+^-only cells out of the total electroporated population from an average of 30.4% to 45.6% when comparing the group with px458::GFP to the group with Crxe1/px458::GFP. The proportion of RXRG and GFP-only double-positive cells out of the total GFP^+^ population increased from an average of 8.59% to 19.58%.

## Discussion

CrxE1 activity was first predicted to be photoreceptor-specific based on in silico studies using mouse DNA sequence,^9^ and the sequence was shown to be active in this population. However, it was later characterized in chick and mouse retina tissue ex vivo, and while it showed the highest levels of activity in photoreceptors in both mouse and chick, it was also shown to be active in rod bipolar cells in mouse.^3^ In the current study, the degree of specificity of this element in the mouse retina was analyzed in vivo for the first time.

As early as p2, there was an observable difference between the localization of CrxE1-positive cells compared to cells reported on by the broadly active promoter CAG (Fig. 1B). The *Crx* gene is highly expressed in precursor and developing photoreceptor cells, and this may indicate that the CrxE1 element is at least in part responsible for this early activity. While these cells at p2 were not stained with RHODOPSIN, the proportion of cells marked only by the CrxE1 reporter and not CAG was consistent with the proportion of CrxE1-only cells that were also RHODOPSIN positive at later time points (Figs. 1B–E). While it is possible that some of the p2 cells were precursor cells that would have become bipolar cells, this should only represent a small portion of the CrxE1 cells. While *Crx* gene expression was also seen to a lesser degree in bipolar cells, the in vivo activity of the CrxE1 element was highly specific to photoreceptors. By p8, RHODOPSIN protein expression delineated the ONL, and at this developmental time point, CrxE1 activity was shown to be specific to rod photoreceptors in the electroporated population. At this time point, we observed 23.9% of CAG::mCherry-positive cells to be in the INL. Considering this proportion decreased throughout development, this is consistent with the approximately 20% of INL cells in the electroporated population that had been observed in the p10 rat retina.^1^ By p14, the delineation between the INL and the ONL was most readily observable, and CrxE1 activity was shown to be consistently and exclusively in the ONL in mouse at this time point. The specific difference between the ex vivo activity of CrxE1 in some bipolar cells^3^ and the in vivo exclusion of activity in these cells is of note. There may be mechanisms by which CrxE1 activity is silenced in bipolar cells in the in vivo environment, or the very small number of cells located outside of the ONL may in fact be bipolar cells and are perhaps labeled in smaller numbers or less robustly than in the ex vivo retina preparation. The possibility that the CrxE1 element turns on transiently or at very low levels in bipolar cells may reduce its utility as a photoreceptor-specific lineage-tracing element. Its utility as a faithful lineage-tracing element for *Crx* gene expression also remains untested.

Notably, there was a difference in the proportion of CrxE1^+^ cells to CAG^+^ cells as a function of time point, especially at p2. This is likely because there were still multipotent RPCs targeted by CAG at p2, but by p8, this population had terminally divided to form postmitotic cells, which may or may not have strongly activated CAG or the CrxE1 element depending on cell subtype. The higher standard deviation of this proportion seen at p2 may similarly be due to the position of the electroporated area relative to the center of the retina. Sections of the retina that are more peripheral will be lagging in the developmental stage and potentially still have more RPCs.

Many of the cells targeted by the CrxE1 element can also be targeted by ubiquitous promoters, as shown in Figure 1. The strengths of using broadly active promoters include targeting a large number of cells, as well as intentionally driving gene expression in multiple cell subtypes. However, one negative impact of this approach is that the cells that are targeted may not be enriched for the cell subtype of interest. Importantly, CAG appears to be more sparsely active in newborn photoreceptor precursor cells. As previously stated, *Crx* transcription begins very early in newborn photoreceptor cells, and we observed CrxE1 activity as early as p2 in a significant portion of cells where CAG was not active.

The CrxE1 element, while highly specific to photoreceptors, is active in fewer cells than the CAG promoter, even in the ONL. Thus, it was important to determine whether a plasmid containing both a ubiquitous promoter and the CrxE1 element could increase the number of photoreceptors that are normally targeted by CAG. To generate mRNAs utilizing CrxE1 in a plasmid, transcription must maintain an efficient start site and continue uninhibited through the intended open reading frame of a target sequence. Thus, the position and orientation of each promoter or enhancer sequence should be tested to determine efficient plasmid activity. Qualitative observations revealed clear differences in reporter activity based on the insertion points of CrxE1 into CAG::GFP (Figs. 2A–F). Despite the fact that promoters such as CAG are assumed to be broadly active in all cells, the results in this study indicate that incorporation of an additional cell subtype–specific enhancer can increase overall activity of these elements. While in this study, we strictly refer to increased activity based on the number of cells targeted, there also appear to be changes in the amount or timing of transcriptional activity within a given cell. It is critical to point out that the goal was not to create a vector that specifically targeted photoreceptors. Given that ubiquitous promoters are still useful components in targeting some photoreceptors, we aimed to enrich for photoreceptor targeting by adding the CrxE1 element to ubiquitous promoter-based targeting. To this end, we chose photoreceptor-specific genes for our gain-of-function and loss-of-function experiments. While CAG-based transcription will still allow the vectors to target other cell types, the focus was on increasing vector activity in photoreceptors within this larger retinal population through the addition of the CrxE1 element.

The ability of *Onecut1* overexpression at p0 in mouse to induce ThrbCRM1 activity by p14 is easily quantifiable using a previously described ThrbCRM1 reporter. Reporter activity of ThrbCRM1, when testing the CrxE1/CAG::OC1 plasmid, increased proportionally with the number of cells targeted by CrxE1 alone compared to CAG (Figs. 1 and 3). We observed an average of 14.1% CrxE1^+^-only cells across developmental time points (Supplementary Table S1). At p14, we also observed a 14.6% increase in ThrbCRM1::TdT cells when using the CrxE1-modified overexpression plasmid compared to the CAG::OC1 phenotype (Supplementary Table S2). This represents a 36% increase over baseline in electroporated cells that activate the ThrbCRM1 reporter. Similarly, in the loss-of-function experiments, we also observed a 14.6% increase in S-OPSIN^+^ electroporated cells at p21 when comparing the CrxE1-modified Cas9 plasmid to the EF1A::Cas9 phenotype, which represents a 42% increase over baseline in the cells with a *Nrl* loss-of-function phenotype (Supplementary Table S3). These two sets of experiments employed different plasmid DNA backbone sequences, and one relied on Cas9 activity and single-guide RNA efficiency while the other did not. The similarity between the change in the percentage of phenotypic cells in this case was not due to an exceptionally low standard deviation in either set of experiments but rather a uniquely common average of nine technical replicates across three biological samples for each. Nevertheless, we can conclude that the similar increase in phenotype is likely directly related to the number of cells that are targeted by the CrxE1 element in both settings.

These data indicate to us that the CrxE1 element drives transcription more readily than CAG in a certain cell type at a certain developmental stage. While any progenitor cell dividing at p0 could be electroporated and eventually activate the CAG promoter, only cells undergoing their terminal division into photoreceptor precursor cells appear to immediately activate the CrxE1 element. As previously stated, the proportional increase in targeting when using the CrxE1 element with CAG compared to CAG alone closely recapitulated the proportional increase in phenotype changes in our gain-of-function and loss-of-function experiments. This leads us to believe that not only will using the CrxE1 element enrich for early photoreceptor cell targeting but that photoreceptor fate-changing manipulations are more efficacious when targeting cells in this developmental stage. This may be related to the early activity of CrxE1 in the context of cell birth. These cells, which remain in the ONL throughout postnatal development, are normally developing rod photoreceptors. However, these photoreceptors are at a stage in their development during which many of them can be manipulated starting at p0 to repress the rod program and instead produce cone-associated proteins RXRG and S-OPSIN.^10^^,^^11^

This increase in phenotypic penetrance is critical not only to this research but also to any other experiments that target developing photoreceptors. This may or may not include experiments that employ other delivery methods beyond plasmid electroporation such as adeno-associated viruses or lentiviruses. Depending on the function of one's gene of interest, this increase in targeting could influence whether a novel phenotype is readily detectable or not. Additionally, in experiments seeking to specifically target rod cells, without affecting any other cell type, we can utilize plasmids that do not contain any ubiquitous promoter and instead only use the CrxE1 element.

We also observe that the CrxE1 element is active during cone formation and can be used to enhance targeting of developing cone photoreceptors ex vivo at E13.5. It is important to point out that in our own previous experiments, the CrxE1 element showed activity in a portion of bipolar cells by ex vivo assay, whereas by in vivo assay, we observed little to no activity outside of the ONL. While ex vivo experimentation has limitations to recapitulating the in vivo environment, these ex vivo data from embryonic time points indicate that the CrxE1 element is likely active in developing cones and could be effective in experiments targeting cone-associated gene regulatory networks.

Importantly, one of the intended outcomes of this study was to interrogate the possibility of enhancing previously studied photoreceptor fate change phenotypes. As previously stated, cells targeted by the CrxE1 element at p0 are poised to respond to photoreceptor-specific transcription factor manipulations to genes such as *Onecut1* and *Nrl*. These results directly apply to photoreceptor generation research and other translational research opportunities regarding the treatment of blinding diseases. If more photoreceptor precursor cells in human organoid and stem cell–based models can be transformed into cones, these manipulations could become the basis for more effective cell transplant treatments. The reporter studies here within show that the CrxE1 element is strongly active in rod photoreceptor cells, including at an early stage in their development. Furthermore, the data reveal that conducting gain-of-function or loss-of-function experiments with this element in addition to a broadly active promoter can lead to changes in gene expression patterns that exceed what has been previously reported. The observed increase in the proportion of phenotypic cells reemphasizes the importance of cell subtype–specific targeting. This phenomenon requires further study and will be the topic of future investigations.

## Supplementary Material

Supplement 1

Supplement 2

Supplement 3

Supplement 4

Supplement 5

Supplement 6

Supplement 7
