# Supplementary material for: Enhanced Transcriptional Activation in Developing Mouse Photoreceptors
Source: Invest Ophthalmol Vis Sci. 2025 Jan 24;66(1):54. doi: 10.1167/iovs.66.1.54 (PMC11760266; doi:10.1167/iovs.66.1.54)
Supplement: Supplement 1 [file iovs-66-1-54_s001.pdf]

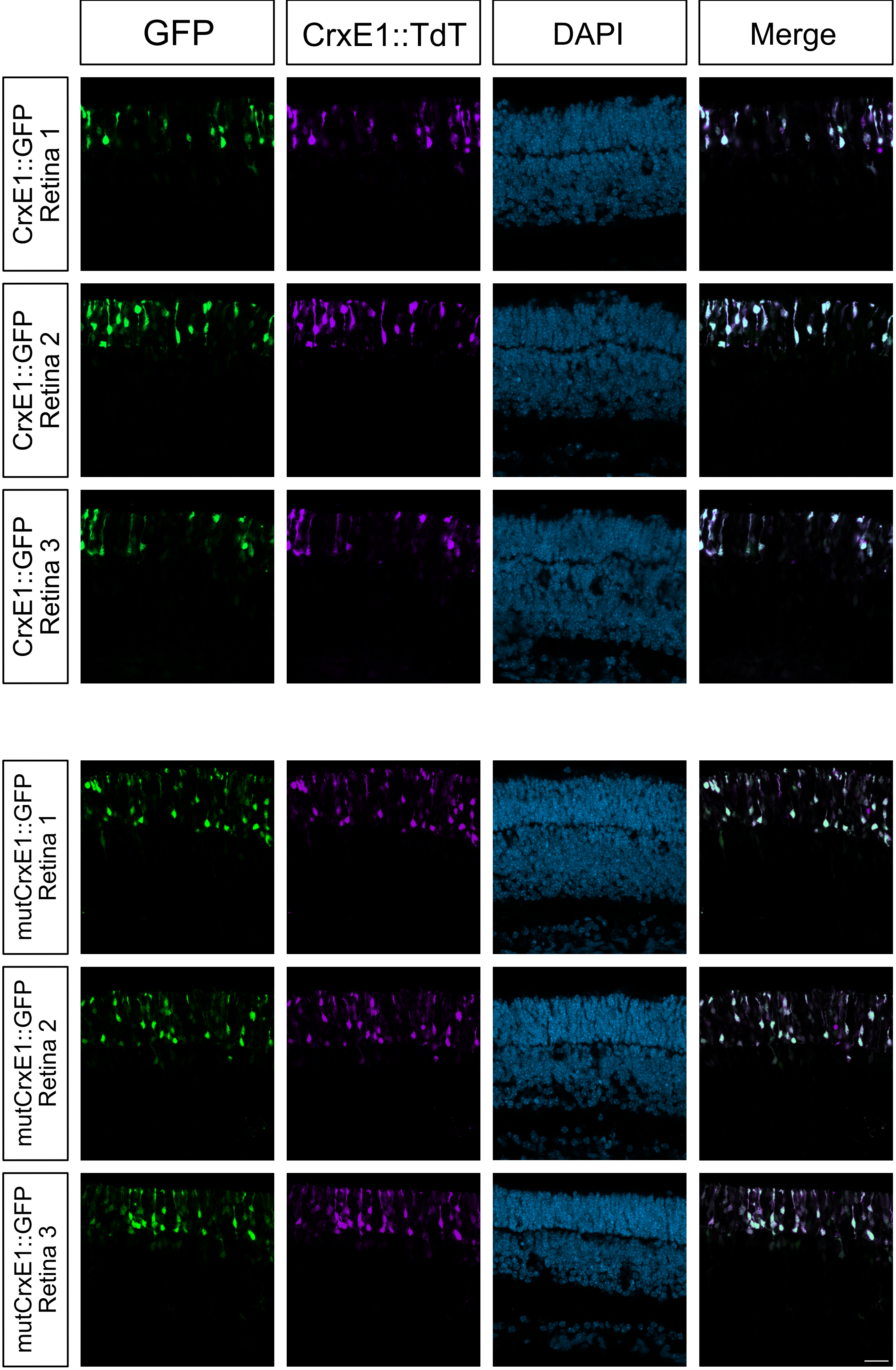

GFP
CrxE1::TdT
DAPI
Merge
CrxE1::GFP Retina 1
CrxE1::GFP Retina 2
CrxE1::GFP Retina 3
mutCrxE1::GFP Retina 1
mutCrxE1::GFP Retina 2
mutCrxE1::GFP Retina 3

Supplemental Figure 1. Confocal microscopy of retinal sections electroporated in vivo at p0 with CrxE1::TdT and either CrxE1::GFP or mutantCrxE1::GFP (mutCrxE1::GFP). Retinae were processed at p8 and sections labeled with DAPI. Representative z-projections are shown with the developing ONL at the top of the section. Columns from left to right are GFP epifluorescence, TdT epifluorescence, DAPI staining in blue, and a merge of the GFP and TdT reporter channels. Scale bar is 20μm and applies to all panels.
